# Supplementary material for: Comparative multiomics analysis of cell physiological state after culture in a basket bioreactor
Source: Sci Rep. 2022 Nov 23;12:20161. doi: 10.1038/s41598-022-24687-4 (PMC9686226; doi:10.1038/s41598-022-24687-4)
Supplement: Supplementary file 1 — Supplementary Information 1. [file 41598_2022_24687_MOESM1_ESM.zip › raw data/Proteomic raw data/4.DiffExprAnalysis/EG.vs.CG/EG.vs.CG.heatmap.detail2.pdf]

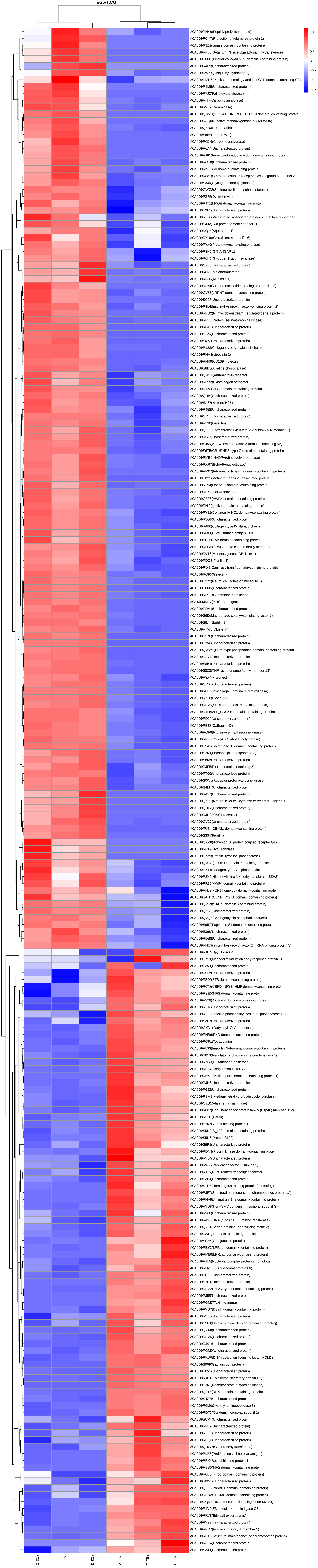
EG.vs.CG
1.5
1
0.5
0
−0.5
−1
−1.5
A0A0D9RNY6(Peptidylprolyl isomerase)
A0A0D9RCY7(Protection of telomeres protein 1)
A0A0D9R3Z2(Lipase domain−containing protein)
A0A0D9RFB3(Beta−1,4−N−acetylgalactosaminyltransferase)
A0A0D9S6M1(Fibrillar collagen NC1 domain−containing protein)
A0A0D9R469(Uncharacterized protein)
A0A0D9RWK4(Ubiquitinyl hydrolase 1)
A0A0D9RMP6(Pleckstrin homology and RhoGEF domain containing G3)
A0A0D9R4M5(Uncharacterized protein)
A0A0D9RIT7(Carbonic anhydrase)
A0A0D9R7J2(Palmitoyltransferase)
A0A0D9RNZ2(Ceramidase)
A0A0D9QWZ8(G_PROTEIN_RECEP_F3_4 domain−containing protein)
A0A0D9RAQ0(Putative monooxygenase p33MONOX)
A0A0D9QZU3(Tetraspanin)
A0A0D9S6E9(Protein Wnt)
A0A0D9QX66(Carbonic anhydrase)
A0A0D9R6A4(Uncharacterized protein)
A0A0D9RJ61(Ferric oxidoreductase domain−containing protein)
A0A0D9RQT0(Uncharacterized protein)
A0A0D9RKG1(IMI domain−containing protein)
A0A0D9R8B1(G protein−coupled receptor class C group 5 member A)
A0A0D9S336(Glycogen [starch] synthase)
A0A0D9QWC2(Sphingomyelin phosphodiesterase)
A0A0D9RZ75(Dystrobrevin)
A0A0D9RJT1(MAGE domain−containing protein)
A0A0D9S9E2(Uncharacterized protein)
A0A0D9RZ80(Microtubule−associated protein RP/EB family member 2)
A0A0D9S432(Two pore segment channel 1)
A0A0D9RQJ5(Aquaporin−1)
A0A0D9RXU0(Growth arrest specific 6)
A0A0D9RYA9(Protein−tyrosine−phosphatase)
A0A0D9R3N7(GIT ArfGAP 1)
A0A0D9R6H1(Glycogen [starch] synthase)
A0A0D9QX48(Uncharacterized protein)
A0A0D9R8N8(Melanotransferrin)
A0A0D9RBB5(Muskelin 1)
A0A0D9RL I9(Guanine nucleotide−binding protein−like 3)
A0A0D9QV56(LRRNT domain−containing protein)
A0A0D9SC08(Uncharacterized protein)
A0A0D9R9L4(Insulin−like growth factor−binding protein 2)
A0A0D9RBU2(N−myc downstream−regulated gene 1 protein)
A0A0D9RPF3(Protein−serine/threonine kinase)
A0A0D9RGE1(Uncharacterized protein)
A0A0D9S126(Uncharacterized protein)
A0A0D9S0Y3(Uncharacterized protein)
A0A0D9R128(Collagen type VIII alpha 1 chain)
A0A0D9RNH8(Lipocalin 2)
A0A0D9RNK9(CD109 molecule)
A0A0D9S8B5(Alkaline phosphatase)
A0A0D9QWT4(Anthrax toxin receptor)
A0A0D9RRB2(Plasminogen activator)
A0A0D9R1Z8(MFS domain−containing protein)
A0A0D9QUI4(Uncharacterized protein)
A0A0D9SA87(Histone H2B)
A0A0D9R458(Uncharacterized protein)
A0A0D9QV40(Uncharacterized protein)
A0A0D9R590(Galectin)
A0A0D9QXG6(Cytochrome P450 family 2 subfamily R member 1)
A0A0D9RCI9(Uncharacterized protein)
A0A0D9S455(von Willebrand factor A domain containing 5A)
A0A0D9S6T5(GB1/RHO3−type G domain−containing protein)
A0A0D9RMB0(NADP−retinol dehydrogenase)
A0A0D9RXR7(Ecto−5−nucleotidase)
A0A0D9RM07(Fibronectin type−III domain−containing protein)
A0A0D9S8Y2(Matrix remodeling−associated protein 8)
A0A0D9R294(Lipase_3 domain−containing protein)
A0A0D9RFK1(Calsyntenin 2)
A0A0D9QZZ6(VWFA domain−containing protein)
A0A0D9RNI2(Ig−like domain−containing protein)
A0A0D9RY12(Collagen IV NC1 domain−containing protein)
A0A0D9R3G8(Uncharacterized protein)
A0A0D9R486(Collagen type IV alpha 3 chain)
A0A0D9RQI3(B−cell surface antigen CD40)
A0A0D9SE86(AAA domain−containing protein)
A0A0D9RHR6(ARVCF delta catenin family member)
A0A0D9RVT6(Monooxygenase DBH like 1)
A0A0D9RDQ3(Fibrillin 1)
A0A0D9RIK3(Carn_acyltransf domain−containing protein)
A0A0D9RQ93(Galectin)
A0A0D9S2Z2(Neural cell adhesion molecule 1)
A0A0D9S6M6(Uncharacterized protein)
A0A0D9RRE1(Glutathione peroxidase)
A0A1J0MKR7(MHC IB antigen)
A0A0D9RRA4(Uncharacterized protein)
A0A0D9S6I0(Macrophage colony−stimulating factor 1)
A0A0D9S6J4(Sortilin 1)
A0A0D9RTW6(Clusterin)
A0A0D9S1Z0(Uncharacterized protein)
A0A0D9S3V0(Uncharacterized protein)
A0A0D9QWW1(PPM−type phosphatase domain−containing protein)
A0A0D9R2V7(Uncharacterized protein)
A0A0D9S8B1(Uncharacterized protein)
A0A0D9S8Z3(TNF receptor superfamily member 18)
A0A0D9RX44(Fibronectin)
A0A0D9QVG1(Uncharacterized protein)
A0A0D9R8E6(Procollagen−proline 4−dioxygenase)
A0A0D9R710(Plexin A1)
A0A0D9REV5(SERPIN domain−containing protein)
A0A0D9R4L5(ZnF_CDGSH domain−containing protein)
A0A0D9RD29(Uncharacterized protein)
A0A0D9RBZ8(Cathepsin D)
A0A0D9RQF9(Protein−serine/threonine kinase)
A0A0D9RK80(Poly [ADP−ribose] polymerase)
A0A0D9S1A5(Lactamase_B domain−containing protein)
A0A0D9S760(Phospholipid phosphatase 3)
A0A0D9S8K6(Uncharacterized protein)
A0A0D9RJF5(Plexin domain containing 2)
A0A0D9RT09(Uncharacterized protein)
A0A0D9S0W1(Receptor protein−tyrosine kinase)
A0A0D9S4M4(Uncharacterized protein)
A0A0D9R457(Uncharacterized protein)
A0A0D9QXP1(Natural killer cell cytotoxicity receptor 3 ligand 1)
A0A0D9QUL2(Uncharacterized protein)
A0A0D9RJD8(KISS1 receptor)
A0A0D9QV27(Uncharacterized protein)
A0A0D9RJ34(CBM21 domain−containing protein)
A0A0D9S334(Ferritin)
A0A0D9QXV0(Adhesion G−protein coupled receptor G1)
A0A0D9RP19(Hyaluronidase)
A0A0D9S7Z9(Protein−tyrosine−phosphatase)
A0A0D9QW82(GLOBIN domain−containing protein)
A0A0D9RY11(Collagen type IV alpha 2 chain)
A0A0D9R3X6(Histone−lysine N−methyltransferase EZH2)
A0A0D9R3S(VWFA domain−containing protein)
A0A0D9RHJ9(FCP1 homology domain−containing protein)
A0A0D9SAH4(CENP−V/GFA domain−containing protein)
A0A0D9QVS9(START domain−containing protein)
A0A0D9QX59(Uncharacterized protein)
A0A0D9QVQ6(Sphingomyelin phosphodiesterase)
A0A0D9S957(Peptidase S1 domain−containing protein)
A0A0D9S368(Uncharacterized protein)
A0A0D9RDB8(Uncharacterized protein)
A0A0D9RNC9(Insulin like growth factor 2 mRNA binding protein 3)
A0A0D9RJD4(Dpy−19 like 4)
A0A0D9S718(Mesoderm induction early response protein 1)
A0A0D9S253(Uncharacterized protein)
A0A0D9R9F6(Uncharacterized protein)
A0A0D9R200(BTB domain−containing protein)
A0A0D9R6T8(CBFD_NFYB_HMF domain−containing protein)
A0A0D9RSE0(MFS domain−containing protein)
A0A0D9R329(Aa_trans domain−containing protein)
A0A0D9RZ16(Uncharacterized protein)
A0A0D9RX83(Inactive phosphatidylinositol 3−phosphatase 12)
A0A0D9S2P7(Uncharacterized protein)
A0A0D9QXD1(Fatty acyl−CoA reductase)
A0A0D9RNB6(PKD domain−containing protein)
A0A0D9RQF1(Tetraspanin)
A0A0D9S533(Importin N−terminal domain−containing protein)
A0A0D9S810(Regulator of chromosome condensation 1)
A0A0D9RG70(Glutathione transferase)
A0A0D9RFF4(Coagulation factor V)
A0A0D9RSW0(Motile sperm domain−containing protein 1)
A0A0D9RJH6(Uncharacterized protein)
A0A0D9R633(Uncharacterized protein)
A0A0D9RSM3(Methenyltetrahydrofolate cyclohydrolase)
A0A0D9QZS1(Alanine transaminase)
A0A0D9RB87(DnaJ heat shock protein family (Hsp40) member B12)
A0A0D9RFU7(Derlin)
A0A0D9S7K7(Y−box binding protein 1)
A0A0D9S5N3(S_100 domain−containing protein)
A0A0D9S5N6(Protein S100)
A0A0D9S9P1(Uncharacterized protein)
A0A0D9RDN3(Protein kinase domain−containing protein)
A0A0D9RY84(Uncharacterized protein)
A0A0D9RWI0(Replication factor C subunit 1)
A0A0D9RJ75(Runt−related transcription factor)
A0A0D9S2L0(Uncharacterized protein)
A0A0D9S1R5(Homologous−pairing protein 2 homolog)
A0A0D9RJF7(Structural maintenance of chromosomes protein 1A)
A0A0D9RHA9(Aminotran_1_2 domain−containing protein)
A0A0D9RVD8(Non−SMC condensin I complex subunit G)
A0A0D9R266(Uncharacterized protein)
A0A0D9R4V8(DNA (cytosine−5)−methyltransferase)
A0A0D9QYJ1(Serine/arginine−rich splicing factor 2)
A0A0D9RBJ7(J domain−containing protein)
A0A0D9SCE4(Gap junction protein)
A0A0D9REY3(LRRcap domain−containing protein)
A0A0D9RM50(LRRcap domain−containing protein)
A0A0D9R1L5(Nucleolar complex protein 3 homolog)
A0A0D9RN23(60S ribosomal protein L6)
A0A0D9SDZ3(Uncharacterized protein)
A0A0D9S7U1(Uncharacterized protein)
A0A0D9RFM8(RING−type domain−containing protein)
A0A0D9RJ55(Uncharacterized protein)
A0A0D9RQM7(Taxilin gamma)
A0A0D9RYG7(Death domain−containing protein)
A0A0D9RY85(Uncharacterized protein)
A0A0D9S1L3(Meiotic nuclear division protein 1 homolog)
A0A0D9QYX8(Uncharacterized protein)
A0A0D9REV4(Uncharacterized protein)
A0A0D9R491(Uncharacterized protein)
A0A0D9RQM0(Uncharacterized protein)
A0A0D9RKU0(DNA replication licensing factor MCM3)
A0A0D9S909(Gap junction protein)
A0A0D9S6V2(Uncharacterized protein)
A0A0D9RJC1(Epididymal secretory protein E1)
A0A0D9S361(Receptor protein−tyrosine kinase)
A0A0D9QZT6(RRM domain−containing protein)
A0A0D9S4Z7(Uncharacterized protein)
A0A0D9R0M0(X−prolyl aminopeptidase 3)
A0A0D9RDT3(Condensin complex subunit 1)
A0A0D9SCP4(Uncharacterized protein)
A0A0D9R2B7(Uncharacterized protein)
A0A0D9RXG3(Uncharacterized protein)
A0A0D9REQ6(Uncharacterized protein)
A0A0D9QUN7(Glucuronosyltransferase)
A0A0D9RLR8(Proliferating cell nuclear antigen)
A0A0D9RFI6(Retinol binding protein 1)
A0A0D9RSB0(MFS domain−containing protein)
A0A0D9R0B8(IF rod domain−containing protein)
A0A0D9S0W6(Uncharacterized protein)
A0A0D9QZ96(RanBD1 domain−containing protein)
A0A0D9RED2(THUMP domain−containing protein)
A0A0D9RQN8(DNA replication licensing factor MCM4)
A0A0D9R1V2(E3 ubiquitin−protein ligase CBL)
A0A0D9RRV8(Bile salt export pump)
A0A0D9RYS3(Uncharacterized protein)
A0A0D9RFQ7(Golgin subfamily A member 5)
A0A0D9RFT6(Structural maintenance of chromosomes protein)
A0A0D9RAK4(Uncharacterized protein)
A0A0D9S239(Uncharacterized protein)
PCI_2
PCI_1
PCI_3
PEL_2
PEL_1
PEL_3
